# Renin-Angiotensin-Aldosterone System (RAS) Inhibitors May Suppress the Prevalence of Peripheral Arterial Disease (PAD) in Elderly, Chronic Hemodialysis Patients

**DOI:** 10.7759/cureus.25087

**Published:** 2022-05-17

**Authors:** Ryo Tomaru

**Affiliations:** 1 Nephrology, Medical Corporation Shomikai Sakurashinmachi Clinic, Tokyo, JPN

**Keywords:** pad (peripheral artery disease), ckd (chronic kidney disease), hd (hemodialysis), abi (ankle brachial index), spp (skin perfusion pressure)

## Abstract

According to the hypertension guidelines, calcium antagonists are recommended as antihypertensive drugs for Stage 5 of chronic kidney disease (CKDG5) and late elderly patients, whereas renin-angiotensin-aldosterone system (RAS) inhibitors (RASi) are not recommended. We screened elderly CKDG5D patients at a single outpatient maintenance hemodialysis center for the progression of peripheral arterial disease (PAD) using the ankle-brachial index (ABI) and skin perfusion pressure (SPP) tests, as well as logistic regression analysis, to determine the association between PAD and the treatment with RASi and the association between the treatment with RASi and the need for hospitalization within one year of observation. With the presence of PAD as the explanatory variable and the presence of RASi as the objective variable, the odds ratio was 1.23 (95% confidence interval [CI] 0.37 to 3.82) in univariate analysis. After adjusting for confounding factors (age, gender, and hypertension), the odds ratio in multivariate analysis was 0.83 (95% CI 0.46 to 6.08). The presence or absence of PAD was significantly associated with an odds ratio of 3.24 (p = .04, 95% CI 1.0 to 10.25) and 4.63 (p = .026, 95% CI 1.20 to 17.84) in univariate and multivariate analyses, respectively, when the outcome was hospitalization at one year, regardless of the presence or absence of RASi. However, in univariate analysis, the odds ratio was 1.23 (95% CI 0.37 to 3.82) with RASi status as the explanatory variable and one-year hospitalization as the objective variable. After adjusting for confounders, the odds ratio in multivariate analysis was 0.83 (95% CI 0.46 to 6.08). Although further large-scale, multicenter studies are needed to establish the evidence, our results suggest that RASi treatment may have a suppressive effect on the prevalence of PAD and the need for hospitalization in elderly CKDG5 dialysis (CKDG5D) patients.

## Introduction

General hypertension guidelines were published in 2019 [1] as generic guidelines. In these guidelines and the chronic kidney disease (CKD) G3b‒5 medical care guidelines [2] of the Japanese Society of Nephrology, a calcium-receptor antagonist is the first choice as an antihypertensive drug for the late elderly (late-stage elderly over 75 years old) with CKD group 5 dialysis (CKDG5D), and renin-angiotensin-aldosterone system inhibitors (RASi), including angiotensin receptor blockers (ARBs), are not recommended. However, in CKDG5D, there have been many reports that ARBs demonstrate effective antihypertensive and protective effects against cardiovascular events in CKDG5D patients [3-6]. However, a meta-analysis paper integrating the aforementioned studies did not find any evidence of a cardiovascular event suppression effect in CKDG5D patients but concluded that further comparison of study results may be necessary [7]. The antihypertensive and improvement-of-prognosis effects of these RASi are controversial in the patient population [8-11], especially in elderly patients undergoing hemodialysis (HD). In this study, we examined whether the use or nonuse of RASi is beneficial for elderly CKDG5D patients based on the progress of their PAD.

This article was at the 43rd Annual Meeting of the Japanese Society of Hypertension (Okinawa Convention Center, Okinawa, Japan).

## Materials and methods

Patients

The study was conducted from December 2019 to April 2020 and included 70 patients undergoing chronic maintenance hemodialysis at a single facility.

They had agreed to undergo cardio-ankle vascular index (CAVI) testing (Blood Pressure Venography System, VaSeraVS-2000 series, ver.03 FukudaDenshi; Tokyo, Japan) and skin perfusion pressure (SPP) testing (PAD4000 ver.2.14, Kaneka, Tokyo, Japan) as screening tests for PAD in both lower limbs.

Methods 

Study Design

This study was an observational study of dialysis patients at a single center and had three research objectives:

1. This was a cross-sectional study to investigate whether the presence or absence of RASi is associated with the presence or absence of PAD. The presence or absence of RASi was used as the explanatory variable and the presence or absence of PAD as the objective variable.

2. This was a retrospective cohort study, in which the presence or absence of RASi was the explanatory variable and the presence or absence of hospitalization from December 2019 to December 2020 was the objective variable. The study was conducted retrospectively using medical records.

3. A logistic regression analysis was performed with the presence or absence of RASi and the presence of PAD as explanatory variables and the occurrence of hospitalization during the year as the objective variable. Age, gender, history of dialysis, pulse pressure, and calcium*phosphorus (Ca*Pi) products (at the start of dialysis) were added as explanatory variables, which were considered to be clinically confounding factors. Univariate and multivariate analyses were performed.

Data Collection

During the same period (December 2019 to May 2020), patients with an ankle-brachial index (ABI) of 0.9 or lower or an SPP of less than 30 on any one CAVI or SPP test were defined as “having PAD,” and the use or nonuse of a RASi was retrospectively determined based on medical records [12].

The CAVI and SPP tests were performed during hemodialysis therapy for all patients. All but two patients underwent regular hemodialysis for three to four hours three times weekly. One patient underwent hemodialysis once a week for three to four hours in combination with peritoneal dialysis, and one patient underwent hemodialysis once a week for three to four hours to keep residual renal function. Each patient’s age, gender, history of dialysis, the presence of diabetes mellitus, and the presence of PAD were noted at the start of the study. The patients were divided into a group treated with a RASi (RASi (+) group) and a group not treated with a RASi (RASi (-) group). The patient’s age and duration of dialysis were investigated in terms of continuous variables and the percentage of patients older than 75 years and the percentage of patients who had undergone dialysis for more than five years. In addition, the following clinical indicators were measured: blood pressure at the start of dialysis, mean blood pressure and pulse pressure (mean blood pressure at the start of dialysis), height, weight (dry weight), body mass index (BMI), geriatric nutrition risk index (GNRI) [13-14], and estimated salt intake, as calculated from pre and post-dialysis blood samples [15-16]. For blood test data, the following monthly serum concentrations recommended by the Japanese Society for Dialysis Therapy were measured. All for serum calcium (corrected value), phosphorus, Ca*Pi products, potassium, creatinine, urea nitrogen, uric acid, albumin, low-density lipoprotein cholesterol (LDL-C), albumin, high-density lipoprotein cholesterol (HDL-C), and triglyceride levels. In the medical record survey, we investigated whether or not the patients had received vitamin D preparations during the same period; whether or not they had taken antiplatelet, anticoagulant, or prostaglandin preparations; and whether or not they had received erythropoietin-stimulating agents (ESAs) during dialysis. Details of the administration of antihypertensive drugs were also investigated in the medical record survey, including whether a RASi was administered at the start of the study, the generic name and daily dose of the RASi drug, and if any antihypertensive drugs other than RASi were taken. From the patients’ medical records during the first year of the study, we investigated whether each patient had died from cardiovascular disease or any other causes, had suffered an onset of cardiovascular disease, or had been hospitalized for illnesses requiring treatment other than a short-term hospitalization for a medical checkup.

Primary, Secondary, and Tertiary Endpoints

In line with the research objectives outlined above:

1. Logistic regression analysis was performed with the presence or absence of PAD as the objective variable and the presence or absence of RASi as the explanatory variable as the primary endpoint. Age, gender, history of dialysis, and presence of hypertension, which were considered to be clinically confounding factors, were added as objective variables. Univariate and multivariate analyses were performed.

2. Logistic regression analysis was performed with the objective variable being the occurrence of hospitalization at one year as the secondary endpoint and the explanatory variable being whether or not the patient was taking RASi. Logistic regression analysis was performed with the objective variable being the occurrence of hospitalization at one year and the explanatory variable being whether or not the patient was taking RASi.

3. We performed univariate and multivariate analyses with the objective variable being the occurrence of hospitalization at one year and the explanatory variables being the presence or absence of RASi and the presence or absence of PAD as composite confounders ( RASi (+) and PAD (+), RASi (+) and PAD (-), RASi (-) and PAD (+), RASi (-) and PAD (-)). Logistic regression analysis was performed. We performed univariate and multivariate analyses by adding the clinically confounding factors age, sex, dialysis history, pulse pressure, and Ca*Pi product as objective variables.

Statistical analysis

Summary statistics were calculated for demographic characteristics and hematological parameters according to treatment group (RASi (+) and RASi (-) groups). We summarized the endpoints on the basis of the treatment group and tested the primary hypothesis using a Kruskal-Wallis test for a numerical variable and a Wilcoxon test for a categorical variable. For the primary and secondary endpoints, we performed a logistic regression analysis to estimate the odds ratio and the 95% confidential interval to calculate p-values. The model included fixed-effect terms for treatment, RASi (+) and RASi (-) group and PAD (+) and PAD (-). P‐values were presented without adjusting for multiple comparisons in an exploratory manner. No statistical sample size calculations were conducted. No imputation for missing data was applied. All statistical analyses were conducted using JMP software version 15.2.1 (SAS Institute, Cary, North Carolina). A p-value of less than .05 was considered statistically significant.

## Results

Patient population

In terms of baseline clinical and laboratory findings, the RASi (+) and RAS (‒) groups had slightly higher serum albumin levels (p = .01), and a higher percentage of patients in the RASi (+) group were receiving ESAs (p = .03) (not described). There were unlikely to be significant differences between the presence or absence of RASi and ESA administration.

Twenty-eight patients (41.4%) were judged to have PAD by the CAVI and SPP tests, and 42 patients (58.6%) were judged to have no PAD. There was a significant correlation between the presence of PAD (+) and PAD (‒) and patient age of more than 75 years and less than 74 years, respectively (Fisher’s exact probability test; χ2 =13.7, p = .0002).

Of the RASi (+) patients, about 90% were taking ARBs. Of the patients taking ARBs, 5.7% took a low dose, 12.9% took a moderate dose, and 11.4% had a high dose (for each drug, the maximum daily dose was defined as "high dose," the moderate dose as "moderate dose,” and the minimum dose as "low dose.” In terms of the number of antihypertensive medications taken (including ARBs), the largest proportion of patients (28.6%) took two or three medications, followed by 24.3% who took one medication. No medication was taken in 11.4% of patients, and four or five medications were taken in 5.7% and 1.4% of patients, respectively.

The most common type of antihypertensive medication, other than ACEi and ARBs, was calcium antagonists (67.1%), followed by beta-blockers (45.7%), diuretics (30%), alpha-blockers (24.3%), and others (1.4%).

Primary endpoints

When the presence or absence of PAD was determined as a target variable and the use or nonuse of RASi as an explanatory variable, logistic regression analysis was performed for univariate analysis, the odds ratio was 1.23 (95% CI 0.37 to 3.82). As a confounding factor, when adjusted according to age, sex, and the presence of hypertension, the odds ratio was 0.83 in multivariate analysis (95% CI 0.46 to 6.08) (Table 1).

**Table 1:** Correlation by logistic regression analysis with the presence/absence of PAD and the presence/absence of RASi Note: Logistic regression analysis was performed with the presence/absence of PAD as the objective variable, and the usage of RASi, age, sex, dialysis history, and hypertension as the explanatory variables. Univariate and multivariate analyses were evaluated, respectively. PAD: peripheral arterial disease; RASi: renin-angiotensin-aldosterone system inhibitors

		Univariate			Multivariate		
		Odds ratio	p-value	95% C.I.	Odds ratio	p-value	95% C.I.
RASi	+	0.89	0.83	0.30-3.52	0.74	0.7	0.21-2.44
	-	1					
Age	≧75years	6.76	0.0002	2.41-20.54	8.5	<0.0001	2.81-29.59
	<75years	1					
Sex	F	1.69	0.35	0.22-1.71	2.0	<0.0001	0.63-6.76
	M	1					
Duration of Dialysis	≧5years	1.1	0.85	0.42-2.88	1.73	0.34	0.56-5.77
	<5years	1					
Hypertension	≧140/90mmHg	0.73	0.62	0.22	0.7	0.61	0.17-2.88
	<140/90mmHg	1					

Secondary endpoints

When one-year hospitalization was determined as a target variable and the use or nonuse of RASi as an explanatory variable, logistic regression analysis was performed. The odds ratio was 1.23 (95% CI 0.37 to 3.82) in univariate analysis for one-year hospitalization, and 0.73 in a multivariate analysis adjusted by age, sex, presence of hypertension, and duration of dialysis as confounding variables (95% CI 0.21 to 2.44) (Table 2). On the other hand, regardless of the use or nonuse of a RASi, the presence or absence of PAD was 3.24 (p = .04, 95% CI 1.0 to 10.25) for univariate analysis and 4.63 (p = .026, 95% CI 1.20 to 17.84) for multivariate analysis, assuming hospitalization within one year as an outcome, which demonstrated a significantly higher ratio (Table 3).

**Table 2:** Correlation by logistic regression analysis with hospitalization and the presence/absence of RASi Note: Logistic regression analysis was performed with hospitalization as the objective variable, and the usage of RASi, age, sex, dialysis history, and hypertension as the explanatory variables. Univariate and multivariate analyses were evaluated, respectively. RASi: renin-angiotensin-aldosterone system inhibitors

		Univariate			Multivariate		
		Odds ratio	p-value	95% C.I.	Odds ratio	p-value	95% C.I.
RASi	+	1.23	0.72	0.37-3.82	1.16	0.81	0.34-3.73
	-	1					
Age	≧75years	0.87	0.8	0.28-2.57	0.99	0.99	0.31-3.73
	<75years	1					
Sex	F	1.43	0.7	0.23-2.20	1.3	0.62	0.41-4.20
	M	1					
Duration of Dialysis	≧5years	1.98	0.22	0.67-6.16	1.92	0.25	0.64-6.13
	<5years	1					
Hypertension	≧140/90mmHg	0.73	0.65	0.20-3.03	0.76	0.7	0.20-3.26
	<140/90mmHg	1					

**Table 3:** Correlation by logistic regression analysis with hospitalization and the presence/absence of PAD Note: Logistic regression analysis was performed with hospitalization as the objective variable, and the presence/absence of PAD and PAD-related factors (pulse pressure, Ca*Pi products), age, sex, and dialysis history as the explanatory variables. Univariate and multivariate analyses were evaluated, respectively. The confounding factors were adjusted for age, sex, history of dialysis, pulse pressure (at start of dialysis), and Ca*Pi products. PAD: peripheral arterial disease

		Univariate			Multivariate		
		Odds ratio	p-value	95% C.I.	Odds ratio	p-value	95% C.I.
PAD	+	3.24	0.04	1.08-10.25	4.63	0.026	1.20-17.84
	-	1				

Tertiary endpoints

The odds ratio for RASi (+) and PAD (+) versus RASi (‒) and PAD (‒) was as high as 4.6 for univariate analysis (p = .08, 95% CI 0.84 to 26.70) with the possible confounding factors of age, sex, dialysis history, pulse pressure, and Ca*Pi products. The odds ratio was 5.92 (p = .07, 95% CI 0.85 to 40.96) for multivariate analysis with the possible confounding factors of age, gender, history of dialysis, pulse pressure, and Ca*Pi products. In the PAD (‒) group, the odds ratio for RASi (‒) to RASi (+) was 0.84 (p = .84, 95% CI 0.15 to 5.76), which tended to be slightly lower, though not significant (similar results were obtained in the multivariate analysis). Similarly, in the PAD (+) group, the odds ratio for RASi (‒) to RASi (+) was 1.86 (p = .47, 95% CI 0.25 to 9.24), which was not significantly different; however, it tended to increase clinically (similar results were obtained in the multivariate analysis) (Table 4).

**Table 4:** Correlation by logistic regression analysis with hospitalization and the presence/absence of two factors (RASi and PAD) Note: Logistic regression analysis was performed with hospitalization as the objective variable and with complex factors (RASi (+), PAD (+); RASi (-), PAD (-); RASi (+), PAD(-); RASi (-), PAD (+) as the explanatory variables. The confounding factors were adjusted for age, sex, history of dialysis, pulse pressure (at the start of dialysis), and Ca*Pi products. PAD: peripheral arterial disease; RASi: renin-angiotensin-aldosterone system inhibitors

		Univariate			Multivariate		
		Odds ratio	p-value	95% C.I.	Odds ratio	p-value	95% C.I.
RASi (＋), PAD（＋）		4.6	0.08	0.84-26.70	5.92	0.07	0.85-40.96
RASi（ー), PAD（ー）		1					
RASi（＋), PAD（＋）		1.86	0.47	0.43-10.28	1.53	0.65	0.25-9.24
RASi（ー), PAD（＋）		1					
RASi（＋), PAD（ー）		0.84	0.84	0.11-4.60	0.92	0.93	0.15-5.76
RASi（ー), PAD（ー)		1					

Limitations

In this study, the sample size was insufficient due to insufficient preparation of the preliminary research design. When investigating the causal relationship between "RAS inhibitors" and "PAD" by logistic regression analysis, which is the theme of this study, we selected "age," "gender," "history of dialysis," "pulse pressure," and "Ca*Pi product" as confounders but did not select "presence of diabetes. As a result of insufficient consideration in the sample size design, a clear bias was created between "diabetic" and "non-diabetic," and we thought that a correct evaluation would not be possible so we decided to exclude "presence of diabetes" from the confounders at this time. This was an observational study and further validation by comparative studies with a larger, multicenter, prospective cohort is necessary to build evidence.

## Discussion

Of the eligible patients, 30% were in the RASi (+) group and 70% in the RASi (‒) group so there was a clear majority in the RASi (‒) group. According to a statistical survey by Iseki et al. [17] regarding the Japanese Society for Dialysis Therapy at the end of 2005, among Japanese patients undergoing maintenance hemodialysis three times a week (n = 203,178), 65.7% were taking antihypertensive drugs, with 26.8% using one drug, 24.4% using two drugs, and 14.5% using three or more drugs. Of patients taking ACEi or ARBs, or both, 40.2% were in the RAS (+) group and 25.5% in the RAS (‒) group. Iseki et al. reported on a dialysis study in 2005, during which time hypertension guidelines and treatment guidelines for CKD3‒5 had been established, and the results seemed to be in compliance with the guidelines. The effects of RASi administration on the rates of cardiovascular disease and heart failure and on the rates of death have also been reported [4-6,18-20,21 ]. In the Dialysis Outcomes Practice Pattern Study in Japan (J-DOPPS), RASi had no beneficial effects on cardiovascular events [22]. In our patients, we could not point out any association with cardiovascular disease or cardiovascular death. In CKD, differences in the effects of RASi on the development of cardiovascular disease and heart failure have generally been reported depending on the type and dosage of RASi [23-24]. In our patients, we also investigated the type and dosage of RASi. In patients without CKD, the inhibitory effect of RASi on the development of PAD has been reported since 2008 and, more recently, has also been pointed out in the field of vascular surgery, mainly in general populations [25-26].

Primary endpoints

In this study, we investigated the effect of RASi on PAD in patients with end-stage CKD. As shown in Table 1, RASi (+) had a low univariate odds ratio of 0.89 compared with RASi (‒). In the analysis, the odds ratio was suppressed to 0.74, but the difference was not statistically significant. In this study, ABI and SPP were used to determine the presence or absence of PAD, and if a patient had an ABI of less than 0.9 or an SPP of less than 30, they were considered to be PAD (+). In recent reports on PAD screening by the Japanese Society of Dialysis Therapy, an ABI of less than 0.9 or an SPP of less than 50 was commonly used in dialysis patients [27-30]. The ABI was the same as in the present study, but an SPP of less than 30 was considered PAD (+) in the present study. This may have included some severe cases, but in fact, an SPP of less than 30 or less than 50 is not very different. In general, an SPP value of 30 to 40 is the cutoff value for the diagnosis of PAD in the cardiovascular field; therefore, the lower value was used as the criterion in our study [31-34]. In fact, there was no significant statistical difference between an SPP of less than 30 or less than 50.

Secondary endpoints

Logistic regression analysis was performed with hospitalization as the objective variable and the use or nonuse of RASi as the explanatory variable. It showed that the RASi (+) group was not significantly different from the RASi (‒) group, with an odds ratio of 1.23 (95% CI 0.37 to 3.82) for univariate analysis and 1.16 (95% CI 0.34 to 3.73) for multivariate analysis. There was no significant difference between the two groups (Table 2). The association between PAD (+) and hospitalization without considering RASi, regardless of whether a RASi was being used, when LRA was performed with hospitalization as the objective variable and PAD as the explanatory variable, a PAD (+) patient was significantly more likely to be hospitalized than a PAD (‒) patient, with a univariate odds ratio of 3.24 (95% CI 1.08 to 10.25) and a multivariate odds ratio of 4.63 (95% CI 1.20 to 17.84) (Table 3).

Tertiary endpoints

When RASi (+)/RASi (‒) and PAD (+)/PAD (‒) were set as explanatory variables and hospitalization as the objective variable, RASi (+) was significantly higher than RASi (‒) in the PAD (+) group, with an odds ratio of 1.86 (95% CI 0.43 to 10.28) in univariate analysis and 1.53 (95% CI 0.25 to 9.24) in multivariate analysis, indicating that RASi (+) patients tended to have more hospitalizations. On the other hand, in the PAD (‒) group, the odds ratio of RASi (+) to RASi (‒) was 0.84 (95% CI 0.11 to 4.60) in univariate analysis and 0.92 (95% CI 0.15 to 5.76) in multivariate analysis, indicating that the RASi (+) patients tended to have fewer hospitalizations. Hospitalization was defined as a long-term stay of approximately one month. A short-term hospitalization for medical check-ups and percutaneous transluminal angioplasty (PTA) of vascular access was excluded (Table 4). In the discussion of the design of the sample size, we calculated alpha to be 0.05 and power to be 0.8 in 42% of the RASi group and 35% of the PAD group, and we found that a sample size of 761 patients in each group, for a total of 1522 patients, was necessary.

In our study, the lack of sample size was found to be a limitation; however, the results are likely to be clinically significant, although no statistically significant difference was found, leading to this report [35].

As far as we can tell from a review of the past literature, there is no treatment for PAD at this time that has demonstrated the efficacy of pharmacotherapy for prophylaxis. Although there are no guidelines or case reports, we present this report because further evidence suggests that pharmacotherapy may be useful in the prevention of PAD in hemodialysis patients.

## Conclusions

Although further studies are needed to establish the evidence, it was suggested that RASi may inhibit PAD in CKDG5D patients. In addition, there was a strong possibility that it may prevent hospitalizations.
